# Regulation of Human Endogenous Metabolites by Drug Transporters and Drug Metabolizing Enzymes: An Analysis of Targeted SNP-Metabolite Associations

**DOI:** 10.3390/metabo13020171

**Published:** 2023-01-24

**Authors:** Jeffry C. Granados, Jeramie D. Watrous, Tao Long, Sara Brin Rosenthal, Susan Cheng, Mohit Jain, Sanjay K. Nigam

**Affiliations:** 1 Department of Bioengineering, University of California San Diego, La Jolla, CA 92093, USA; 2 Department of Medicine, University of California San Diego, La Jolla, CA 92093, USA; 3 Department of Pharmacology, University of California San Diego, La Jolla, CA 92093, USA; 4 Center for Computational Biology and Bioinformatics, University of California San Diego, La Jolla, CA 92093, USA; 5 Cedars-Sinai Medical Center, Los Angeles, CA 90048, USA; 6 Department of Pediatrics, University of California San Diego, La Jolla, CA 92093, USA

**Keywords:** transporters, enzymes, ADME, metabolomics, SNPs, pharmacogenomics, fatty acids, eicosanoids, homeostasis, OAT, OATP, MRP

## Abstract

Drug transporters and drug-metabolizing enzymes are primarily known for their role in the absorption, distribution, metabolism, and excretion (ADME) of small molecule drugs, but they also play a key role in handling endogenous metabolites. Recent cross-tissue co-expression network analyses have revealed a “Remote Sensing and Signaling Network” of multispecific, oligo-specific, and monospecific transporters and enzymes involved in endogenous metabolism. This includes many proteins from families involved in ADME (e.g., SLC22, SLCO, ABCC, CYP, UGT). Focusing on the gut−liver−kidney axis, we identified the endogenous metabolites potentially regulated by this network of ~1000 proteins by associating SNPs in these genes with the circulating levels of thousands of small, polar, bioactive metabolites, including free fatty acids, eicosanoids, bile acids, and other signaling metabolites that act in part via G-protein coupled receptors (GPCRs), nuclear receptors, and kinases. We identified 77 genomic loci associated with 7236 unique metabolites. This included metabolites that were associated with multiple, distinct loci, indicating coordinated regulation between multiple genes (including drug transporters and drug-metabolizing enzymes) of specific metabolites. We analyzed existing pharmacogenomic data and noted SNPs implicated in endogenous metabolite handling (e.g., rs4149056 in *SLCO1B1*) also affecting drug ADME. The overall results support the existence of close relationships, via interactions with signaling metabolites, between drug transporters and drug-metabolizing enzymes that are part of the Remote Sensing and Signaling Network, and with GPCRs and nuclear receptors. These analyses highlight the potential for drug−metabolite interactions at the interfaces of the Remote Sensing and Signaling Network and the ADME protein network.

## 1. Introduction

Genome-wide association studies (GWAS) have been used to identify single nucleotide polymorphisms (SNPs) that are linked to phenotypes [1]. The phenotypic traits examined include disease states, drug efficacy, and many others, indicating that GWAS can be used to gain further insight into the genetic causes of many conditions [2,3,4,5]. With the increased generation of large omics datasets, GWAS have also been used to link SNPs to multiple intermediate phenotypes with metabolomics and proteomics [6,7].

While much of the research in this area has focused on identifying differences caused by disease states or other lifestyle factors, GWAS on healthy patients can elucidate the endogenous role of genes by associating specific SNPs to levels of endogenous metabolites. Recent studies have combined GWAS and metabolomics on the plasma and urine of participants to identify potential interactions between proteins and metabolites [8,9,10]. Here, we focused on SNPs in genes of multi-, oligo-, and monospecific transporters and “drug” metabolizing enzymes (DMEs), many of which are best known for their handling of pharmaceutical products, and their associations with circulating endogenous metabolites. The choice of genes was partly influenced by recent data indicating that these multi-, oligo-, and monospecific transporters and enzymes are found in or near hubs in co-expression networks, especially along the gut−liver−kidney axis, suggesting an important endogenous role [11].

Drug transporters and DMEs are among the most studied proteins in pharmacology because of their roles in the ADME (absorption, distribution, metabolism, excretion) of pharmaceutical products [12]. Many of the multispecific drug transporters and DMEs have the capacity to handle structurally diverse drugs, while their more oligo- and (relatively) monospecific counterparts may transport or modify as few as one or two substrates [13,14,15]. GWAS have linked SNPs in these genes to changes in drug toxicity, efficacy, and distribution [16,17].

However, the multispecific nature of these proteins is not limited to pharmaceutical products [18]. Mainly in model organisms but also in humans, endogenous metabolites, including those with well-defined signaling roles, have also been identified as likely in vivo substrates of these proteins (e.g., OAT1, OAT3), often supported by in vitro studies [19]. In GWAS, other results have demonstrated that SNPs in transporter and enzyme genes are associated with endogenous metabolites participating in biochemical pathways, like amino acid catabolism, glycolysis, ketone body metabolism, and others [9,10,20,21,22].

Understanding the full range of endogenous substrates of drug transporters and DMEs can help uncover the physiological metabolic processes that are perturbed when a patient takes drugs. In drug−metabolite interactions (DMI), a drug competes with a metabolite for access to a transporter or enzyme, and thus shifts metabolism by impacting the intracellular and extracellular concentration of the endogenous substrate [23]. Pharmacogenomic studies have focused on the implications of polymorphisms in these genes with respect to drug handling, but the “natural” function of these genes and its potential impact on drug-induced diseases or drug side effects has received comparatively little attention.

The Remote Sensing and Signaling Theory proposes that the primary function of drug transporters and DMEs, together with closely related genes, is to help optimize levels of endogenous metabolites in bodily fluids and tissues by mediating inter-organ and inter-organismal (e.g., gut−microbe−host) communication through small molecule metabolites and signaling molecules [11]. This mechanism, while now experimentally supported in model organisms [24,25,26,27,28], is also supported in humans [29]. Many endogenous metabolites have signaling capabilities that contribute to the regulation of the expression and/or function of other membrane transporters and enzymes by activating nuclear receptors, creating feedback loops [30].

Furthermore, many of these proteins share substrates with one another and are expressed in multiple epithelial tissues, suggesting the possibility of remote communication via these proteins, thereby mediating organ crosstalk [31]. Transporters are regulators of entry (uptake) and exit (efflux) of compounds into the epithelial tissues and body fluids they separate. For example, solute carrier organic (SLCO), solute carrier 22 (SLC22), and ATP-binding cassette subfamily C (ABCC) transporters are expressed in many of the same barrier epithelia tissues, like the proximal tubule (blood−urine), hepatocyte (blood−bile), and choroid plexus (blood−cerebrospinal fluid), and share many common pharmaceutical and endogenous substrates, suggesting that they may be jointly involved in the regulation of these substrates across multiple organs [32]. Indeed, SLC22 and ABCC proteins are among the many “drug transporter” families that were identified as hubs in the aforementioned co-expression gut−liver−kidney network of ~600 proteins—largely consisting of multi-, oligo-, and monospecific transporters, enzymes, and nuclear receptors (including many ADME proteins)—and presented as a preliminary “Remote Sensing and Signaling Network” [11].

The focus of this study is to identify the metabolites and metabolic pathways regulated by these and related proteins in this Remote Sensing and Signaling Network. An additional focus is to determine if evidence for drug−metabolite interactions (DMIs) can be found—given the overlap in proteins of the Remote Sensing and Signaling Network (mediating endogenous small molecule homeostasis) and the ADME protein network (mediating the metabolism and elimination of drugs). The scale of potential DMIs at the level of the major human drug transporters, like organic anion transporter 1(OAT1) and organic anion transporter 3 (OAT3), has recently become evident as well [23].

While a limited number of in vitro cell-based assays and in vivo rodent experiments have been performed to uncover the role of drug-handling proteins in metabolic processes, these experiments can be technically challenging, time-consuming, and labor-intensive—and each has limitations in their application to humans. Virtual screening can aid in this process, but for many proteins, particularly membrane-bound human transporters, determining substrate−transporter interactions has proven to be challenging in part due to the lack of crystal structures [33]. Though lacking in specific mechanisms of action, by using SNP associations with metabolomics data, it is possible to prioritize potential protein−metabolite interactions in humans to evaluate further the possible physiological role of hundreds of genes.

Here, we combined genomic data targeting SNPs in drug transporter, DMEs, and related genes with non-targeted plasma metabolomics of over 2500 patients from the Framingham Offspring Cohort Exam 8 to link SNPs in these genes to the levels of circulating endogenous metabolites. Because the majority of circulating molecules are unknowns, we performed directed, non-targeted LC-MS approaches to specifically capture and assay small, polar, bioactive metabolites, including free fatty acids, eicosanoids and oxylipins, bile acids, fatty acid esters of hydroxy fatty acids, and other related metabolites of known and unknown chemistries. These types of metabolites have been shown to signal via cell surface G-protein coupled receptors (GPCRs) and nuclear receptors and be critical for a host of physiologic processes [34,35].

This work represents a step forward in understanding the individual and combined roles of ADME and other genes in endogenous metabolic processes. Of the several interactions reported here, some have been confirmed by independent in vivo or in vitro experiments, indicating that many novel SNP-metabolite associations likely have a functional protein-ligand relationship. We found metabolites that were linked to multiple SNPs on distinct genomic loci containing genes expressed in different cells and tissues, which raises the possibility of transporter- and/or DME-mediated remote communication via small molecule metabolites. We also analyzed the existing pharmaceutical GWAS to determine DMIs that may occur in patients with genes harboring certain SNPs involved in metabolism. The results indicate that a wide range of DMIs can result at the interfaces of the Remote Sensing and Signaling (protein) network and the ADME (protein) network.

## 2. Materials and Methods

### 2.1. Sample Population

Genotyping was performed on the Framingham Heart Study (FHS) Offspring Cohort Exam 8 (Table 1) [36].

### 2.2. Gene List

The initial gene list for targeted SNP-metabolite associations was constructed based on the Remote Sensing and Signaling Network reported previously based on a co-expression analysis [11]. This network included solute carrier (SLC), ATP-binding cassette (ABC), and several DME families. Among the DME families were subfamilies such as cytochrome P450s (CYPs), uridine 5’-diphospho-glucuronosyltransferases (UGTs), and sulfotransferases (SULTs). This list of multi-, oligo-, and monospecific proteins overlaps considerably with genes known to be involved in the absorption, distribution, metabolism, and excretion (ADME) of drugs. The list was enlarged by considering other transporters and DMEs involved in ADME, as well as related transporters and enzymes based on their roles in ADME, handling of endogenous small molecules, or sequence homologies. In total, this resulted in the consideration of 1131 genes (Supplementary Table S1).

### 2.3. SNP Identification

SNPs were included in the analysis if they were mapped to genes from Supplementary Table S1 using SnpEff [37]. Each SNP was associated with a reference SNP cluster ID (rsID) or a position on a chromosome. Those SNPs with an rsID were present in dbSNP version 151. All SNPs are in hg19 allele reference format.

### 2.4. Imputation

Several genotyping arrays (Affymetrix) were used to identify SNPs for the population. SNPs were imputed using Minimac3. SNPs associated with genes within an initial list of 1131 genes containing drug transporters and drug metabolizing enzymes (Supplementary Table S1) were queried.

### 2.5. Metabolomics Analysis

Metabolomic studies were performed using directed, non-targeted liquid chromatography-mass spectrometry (LC-MS) approaches to specifically capture and assay small polar “bioactive” metabolites. These were deemed to have a higher likelihood of interacting with cell surface receptors involved in signaling. These include free fatty acids, eicosanoids and oxylipins, bile acids, and fatty acid esters of hydroxy fatty acids, among hundreds of unidentified related metabolites [34,35]. Metabolite levels are used as continuous traits with a mean of 0 and standard deviation of 1. Identified metabolites were confirmed through internal standards.

### 2.6. Statistical Analysis

SNP−metabolite *p*-values were determined using linear mixed models (LMM) with an additive genetics model, where 0, 1, and 2 indicate the number of effect alleles for each SNP in the targeted set. The BOLT-LMM algorithm was used to account for age, gender, and other factors [38]. Each metabolite-SNP association had a *p*-value, and only statistically significant associations are reported here. Overall, 673,141 statistically significant SNP-metabolite associations were detected. The *p*-value cutoff for significance was set at 4.9 × 10^−12^.

### 2.7. Genomic Loci

Genomic loci were defined by grouping SNPs that were located within 250,000 base pair windows. SNPs on the same chromosome more than 250,000 base pairs apart were considered to be on different genomic loci. Genes were then mapped to genomic loci if any portion of the gene was within 10,000 base pairs of the genomic locus. For loci without any genes within 10,000 base pairs, the nearest gene was associated with the locus. The GRCh37 build was used for all mapping. Genomic plots were generated using FUMA [39]. Manhattan plots were generated using Assocplots [40].

### 2.8. Tissue-Specific Enrichment

Tissue-specific enrichment for genes within genomic loci was calculated using the TissueEnrich web-based tool (https://tissueenrich.gdcb.iastate.edu, accessed on 3 May 2021) [41]. Supplementary Table S1 was used as the background gene list, and the 178 genes within the genomic loci were used as the input gene list. Tissue expression was determined using the Human Protein Atlas.

### 2.9. Disease and Pharmaceutical Variant Associations

The “Variant and Clinical Annotations”, “Variant, Gene, and Drug Relationship Data”, and “Clinical Variant Data” files were downloaded from the PharmGKB database (https://www.pharmgkb.org/downloads, accessed on 23 August 2022) [42].

## 3. Results

### 3.1. 77 Genomic Loci Are Linked to Circulating Levels of Small, Polar Bioactive Molecules

**Figure 1:**
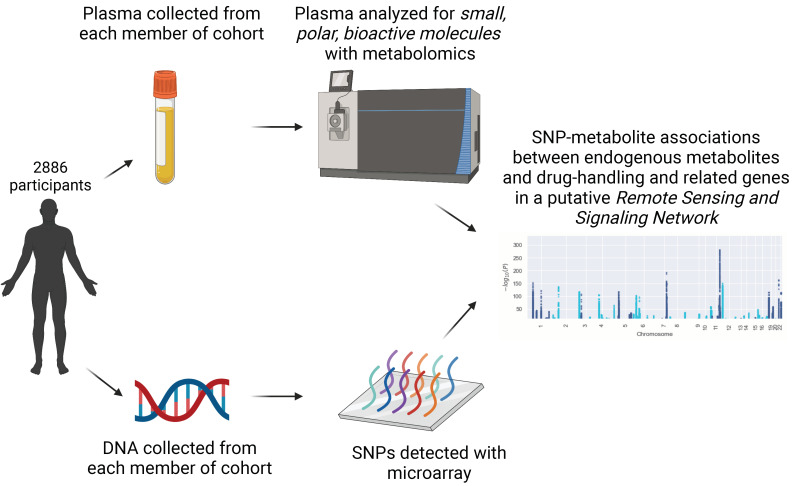
Schematic for data acquisition and subsequent analysis. Plasma was collected from each patient and analyzed by liquid chromatography/mass spectrometry, which sought to capture small, polar, bioactive molecules presumed most likely to be involved in signaling via cell surface and other receptors. DNA was collected from participants and SNPs within a subset of genes were associated with the levels of plasma metabolites.

Plasma and DNA from each participant in the Framingham Offspring Exam 8 Cohort were analyzed to identify relationships between specific genes and endogenous metabolites (Figure 1). We focused on bioactive small, polar molecules, aiming to capture endogenous small molecules that bind receptors involved in signaling. These include eicosanoids, fatty acids, and sex steroids that are known to interact with G-protein coupled receptors (GPCRs) and nuclear receptors (NRs) [43,44].

We analyzed the plasma levels of thousands of unique metabolites and their associations with SNPs contained within a set of 1131 genes. Many of these genes were taken from a previously constructed co-expression network that is believed to reflect their roles mediating endogenous small molecule inter-organ communication, as described in the Remote Sensing and Signaling Theory (RSST). The original list of genes consisted of solute carrier (SLC) transporters, ATP binding cassette (ABC) transporters, DMEs, including CYPs, SULTs, and UGTs, and other drug-related genes that have multi-, oligo-, and monospecific substrate specificity and are expressed in the gut, liver, kidney, and other tissues (Supplementary Table S1). Their known substrates include a wide range of metabolites, signaling molecules, antioxidants, vitamins and cofactors, and gut microbe-derived metabolites. 

**Figure 2:**
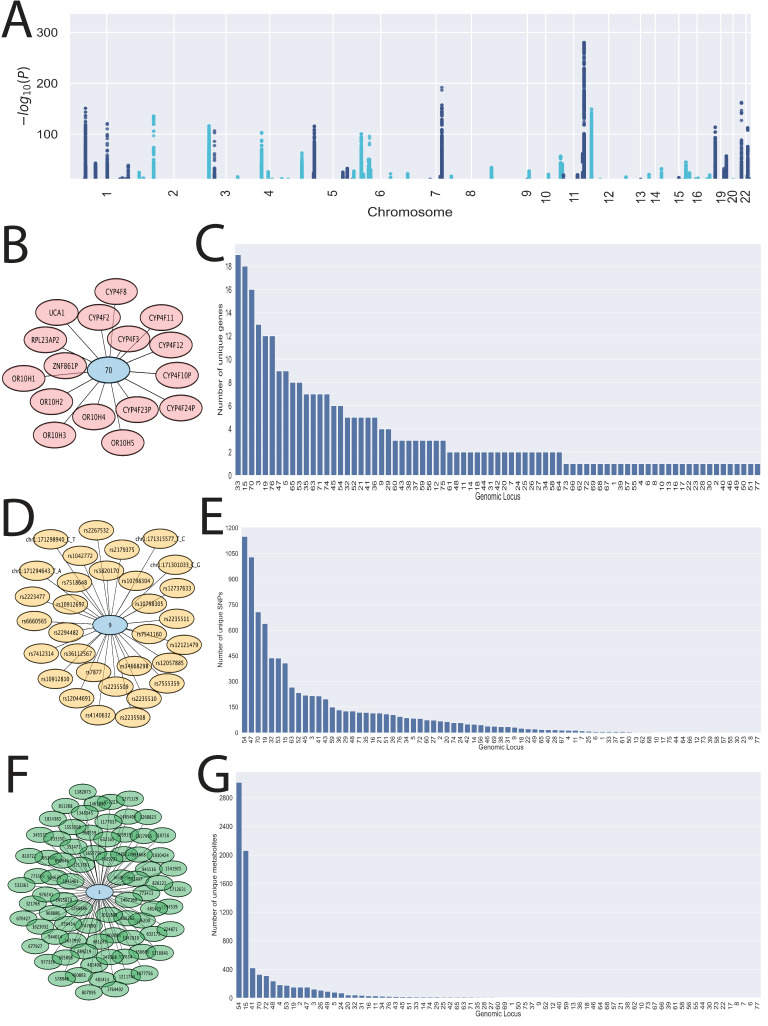
The targeted SNP association study linked SNPs in drug-related genes, like enzymes and transporters, to the circulating levels of small, polar, bioactive molecules. (**A**) Manhattan plot showing the targeted SNP-metabolite associations on the genome. The chromosome and relative genomic location are marked on the x-axis, and the log-scaled *p*-value is marked on the y-axis. Each point in the plot represents an association between the SNP and a measured metabolite. (**B**) Schematic showing the unique genes associated with genomic locus 70, where an edge represents a gene located within or near the genomic locus, as described in the Methods. (**C**) The 77 distinct loci were associated with 284 unique genes, including pseudogenes. Loci were associated with different numbers of unique genes ranging from 19 to 1. (**D**) Schematic showing the unique SNPs associated with genomic locus 9, where an edge represents an SNP located within the genomic locus. (**E**) With respect to SNPs, 8634 unique SNPs were detected, with nearly each loci containing unique SNPs. Some genomic loci, such as genomic loci 47 and 54, were associated with over 1000 distinct SNPs. The number of unique SNPs for each locus ranged from 1149 to 1. (**F**) Schematic showing the unique metabolites associated with genomic locus 5, where an edge represents a statistically significant association. (**G**) 7326 unique, small, polar, bioactive metabolites were measured in the plasma of the participants. Genomic loci 15 and 54 were associated with the highest number of unique metabolites (2059 and 3014, respectively). Unique metabolites associated with each locus ranged from 3014 to 1.

In total, 673,141 statistically significant SNP-metabolite associations were reported, covering 8634 unique SNPs and 7326 unique metabolites (Figure 2A). The surveyed metabolites ranged from mass to charge ratios (M/Z) of 225.110 to 649.3938, and retention time (RT) values ranged from 0.6690834 to 6.988375 s. Each SNP was mapped to a genomic locus based on its position in the GRCh37 build of the human genome, as described in the Methods. We identified 77 distinct genomic loci, which covered 284 unique genes (Supplementary Table S2). Each genomic locus was associated with a different set of genes, SNPs, and metabolites (Figure 2B–G). Genomic locus 15, containing the *UGT1A* genes, and genomic locus 54, containing regions relating to *SLCO1B1*, *SLCO1B3*, *SLCO1B7*, and *SLCO1A2*, were associated with 42% and 33% of the total interactions, respectively. This is consistent with the functions of these genes, as they are known, largely from in vitro work, to be among the most multispecific transporters and enzymes of xenobiotics and metabolites, with dozens of unique substrates. The significant associations are reported in Supplementary Table S3.

### 3.2. Tissue-Specific Enrichment of Genes with SNPs Shows Overrepresentation of Liver Genes

**Figure 3:**
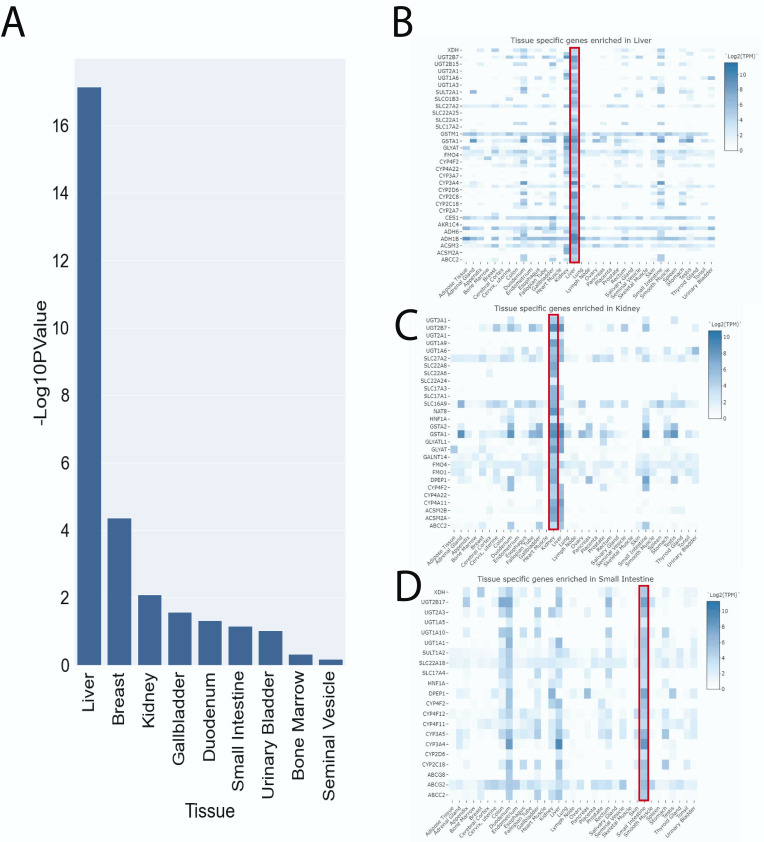
Tissue-specific enrichment reveals over-representation of organs active in ADME (**A**) The genes with significant associations with metabolites were compared to the reference genes listed in Supplementary Table S1. The liver, breast, kidney, gallbladder, duodenum, small intestine, urinary bladder, bone marrow, and seminal vesicle all had significant enrichment. (**B**) Genes enriched in the liver. (**C**) Genes enriched in the kidney. (**D**) Genes enriched in the small intestine.

Of the ~1000 surveyed genes, 178 contained SNPs that were present in our study and significantly associated with at least one endogenous metabolite. Tissue-specific enrichment revealed that the liver, breast, kidney, gallbladder, duodenum, and small intestine were over-represented within these genes (Figure 3A), using the 1131 genes from Supplementary Table S1 as the background set. The liver was the most highly enriched organ (adjusted negative log-scaled *p*-value = 17.1) with 61 tissue-specific genes (Figure 3B). In the pharmaceutical literature, the tissues enriched with these genes are traditionally associated with ADME, with some exceptions. While not traditionally associated with drug ADME, the breast plays a role in the regulation of small molecule metabolites as it contains epithelial tissue that separates the blood and milk and expresses important DMEs of the glutathione S-transferase (GST) and UGT2B families. The next most enriched tissue was the kidney, followed by the gall bladder and intestinal tissues. The 178 metabolite-associated genes were largely enriched in the gut, liver, and kidney, consistent with previously identified roles in remote sensing and signaling of small, polar, bioactive metabolites and signaling molecules across the gut−liver−kidney axis [11] (Figure 3C,D).

### 3.3. Unidentified Metabolites Are Potentially Regulated by Distinct Genomic Loci

**Figure 4:**
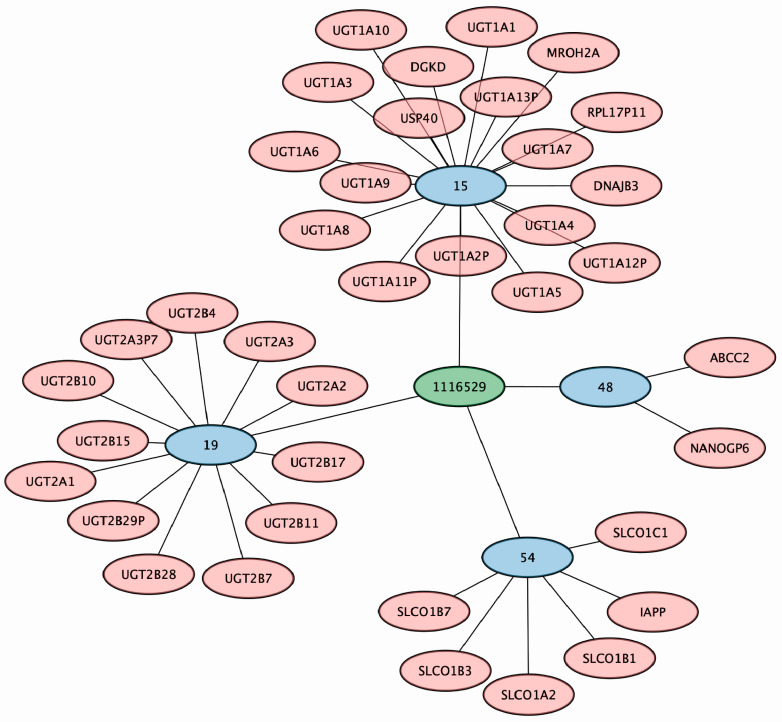
Metabolite 1116529 (MZ: 607.3553, RT: 3.428667) is associated with genomic loci 15, 19, 48, and 54. SNPs within the aforementioned genomic loci are all linked to metabolite 1116529. Genomic locus 15 contains regions relating to the *UGT1A* genes, genomic locus 19 contains regions relating to the *UGT2B7* gene, genomic locus 48 contains regions relating to the *ABCC2* gene, and genomic locus 54 contain regions relating to the *SLCO* genes.

**Figure 5:**
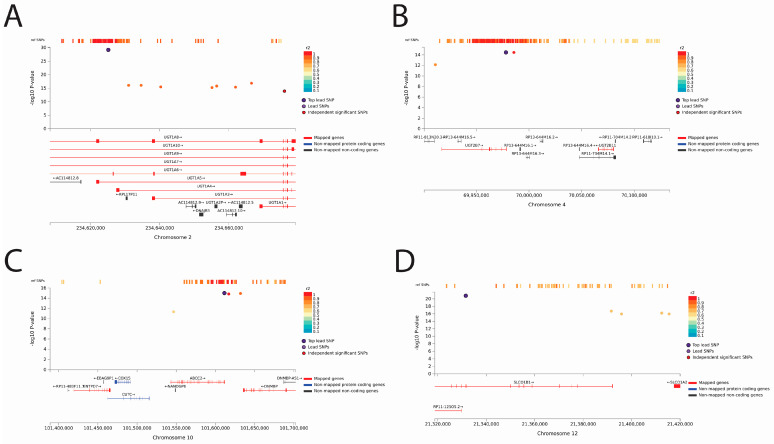
Genomic regions associated with metabolite 1116529, which is associated with four distinct loci. (**A**) Genomic locus 15. (**B**) Genomic locus 19. (**C**) Genomic locus 48. (**D**) Genomic locus 54. In all panels of the figure, the SNPs associated with the levels of circulating metabolite 1116529 are represented by points, where purple points refer to the top lead SNP, and other SNPs are represented by points colored by their r2 value. The r2 value, which represents phenotypic variation, is high in these regions and reference SNPs that have previously been analyzed. The nearest mapped genes are shown below each plot.

Considering that most of the metabolites surveyed were unidentified (chemical identity unknown, but unique mass/charge ratio (MZ) and retention time (RT) combination), we aimed to understand which genomic loci worked collaboratively to regulate or modulate the levels of metabolites rather than focus on the metabolic role of the compound. Depending on the tissue expression and cellular localization of the implicated genes, these could be useful examples in determining potential cases of inter and/or intra-organ communication and lead to a more mechanistic view. We identified five metabolites that were associated with four distinct genomic loci (Table 2). Metabolite 1116529 was the only metabolite not associated with both genomic loci 28 or 29 and was uniquely associated with genomic loci 15, 19, 48, and 54 (Figure 4). Thus, we presented the genomic-regional plots with the implicated SNPs associated with metabolite 1116529 as an example (Figure 5). Metabolite 1116529 was associated with loci containing regions related to the *UGT1A*, *UGT2B7*, *ABCC2*, and *SLCO1B1* genes, which are all multispecific hepatic proteins known to handle metabolites and drugs. Genomic locus 15 includes several genes (*UGT1A6/7/8/9/10*), so it is difficult to associate any single gene with the resulting changes. Nonetheless, the UGT1A genes are primarily expressed in the kidney and liver. Two other implicated genes, UGT2B7 and ABCC2, are also mainly expressed in the kidney and liver, whereas SLCO1B1 is expressed only in the liver.

Even if unidentified, it is still possible to glean some hints of a metabolite’s potential physiological role(s). The function of these proteins (uptake transporter, efflux transporter, and glucuronidation enzymes) and their different sites of expression within the liver (apical plasma membrane, basolateral plasma membrane, and cytosol) and kidney (apical plasma membrane, cytosol) support the view that these proteins work together to regulate the levels of metabolite 1116529 along the liver−kidney axis. We also investigated phenotypes related to the SNPs in these genomic loci from dbSNP and the GWAS catalog and found that the SNPs associated with this metabolite were linked to disorders of bilirubin excretion, serum 25-hydroxyvitamin-D levels, and testosterone levels [45,46]. In addition to this metabolite, there are several other examples of unidentified metabolites associated with multiple genomic loci. With respect to these, 79 metabolites were linked to three distinct loci, including 25 unique combinations; 606 metabolites were linked to two distinct loci; and 6636 metabolites were associated with only 1 genomic locus (Supplementary Figure S1).

### 3.4. Circulating Eicosanoids, Fatty Acids, and Bile Acids Are Impacted by SNPs in 18 Genomic Loci

**Figure 6:**
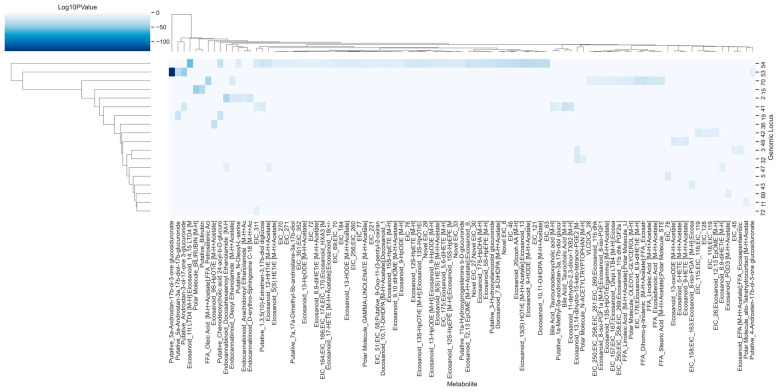
A total of 762 SNP-metabolite associations with identified metabolites were reported, with 18 unique genomic loci and 98 identified metabolites. Values in the heatmap represent the log of the *p*-value of each SNP-metabolite association, with a lower value indicating a stronger relationship between the genomic locus and the metabolite. For improved visualization, the identity of the metabolites in the x-axis has been shortened to include only the first 50 characters. The colorbar showing log-scaled *p*-values has been adjusted to improve visualization, and the highest values go beyond -100. The full names for each identified metabolite are present in Supplementary Table S4.

While all 7326 measured metabolites had a unique metabolite ID, most had not had their chemical identity confirmed. However, 98 metabolites were identified by name, including eicosanoids, fatty acids, and several other signaling molecules. Even in this subset, some metabolites have not been unambiguously identified, but their general class is known. For example, EIC_45 represents a putative eicosanoid [35]. By limiting our analysis to the associations involving these identified metabolites, 762 SNP-metabolite associations were analyzed (Supplementary Table S4). These associations spanned 18 genomic loci, with genomic locus 54 being associated with 62 identified metabolites, the most of any genomic loci surveyed here (Figure 6).

### 3.5. A Putative Eicosanoid Is Independently Associated with SNPs in Phase I and II Drug Metabolism and Transporter Genes

**Figure 7:**
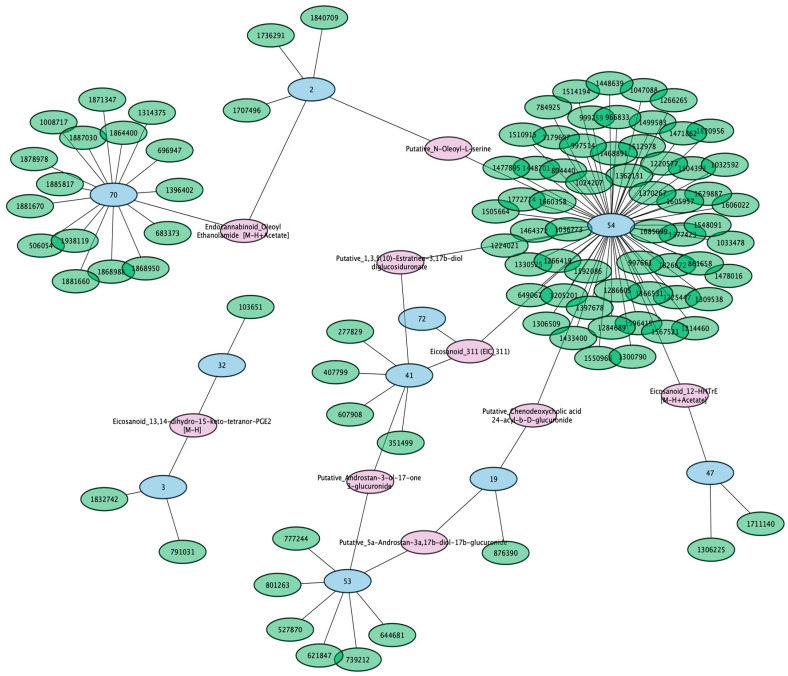
Several identified metabolites were associated with multiple distinct loci. Genomic loci are indicated by blue nodes. All green and purple nodes are identified metabolites. For improved visualization, only those associated with multiple distinct loci are shown in purple and have their identities shown in the figure. The identities for all the green metabolite nodes are listed in Supplementary Table S4. Notably, the eicosanoid EIC_311 is associated with 3 distinct genomic loci, consisting of genomic locus 41 (containing regions relating to the *CYP3A* genes), genomic locus 54 (containing regions relating to the *SLCO* genes), and genomic locus 72 (containing regions relating to the *SULT2A1* gene).

As mentioned in previous sections, we were interested in those metabolites that were associated with multiple genomic loci, as they may be examples of genes involved in inter-organ or intra-organ communication contributing to the systemic levels of particular metabolites. The eicosanoid EIC_311 was the only identified metabolite associated with three unique genomic loci (Genomic loci 41, 54, 72) (Figure 7). These loci contained SNPs in the *CYP3A5*, *SLCO1B1/SLCO1A2*, and *SULT2A1* genetic regions, respectively. These proteins are primarily expressed in the liver and serve critical roles in drug metabolism. CYP3A5 is a Phase I drug-metabolizing enzyme, SULT2A1 is a Phase II drug-metabolizing enzyme, and SLCO1B1/SLCO1A2 are drug transporters (Phase III drug handling), suggesting that these genes may have a combined role in regulating this eicosanoid. Some of these genomic regions have also been linked to blood metabolite levels, urine metabolite levels in chronic kidney disease, and cholelithiasis/cholecystitis [22,47,48]. In addition to EIC_311, eight identified metabolites were also associated with two distinct genomic loci (Table 3).

### 3.6. Conjugated Sex Steroids Are Strongly Associated with SLC22 Genes

**Figure 8:**
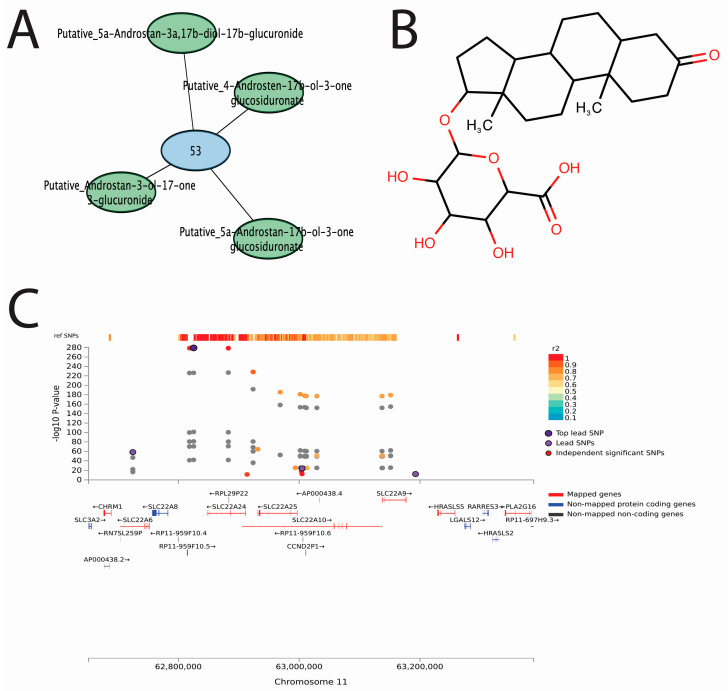
Genomic locus 53, containing SLC22 genes, is associated with conjugated sex steroid hormones. (**A**) The SLC22 genes, *SLC22A6*, *SLC22A8*, *SLC22A9*, *SLC22A10*, *SLC22A24*, and *SLC22A25* are highly associated with circulating levels of 5a-Androstan-17b-ol-3-one glucosiduronate, 4a-Androstan-17b-ol-3-one glucosiduronate, Androstan-3-ol-17-one 3-glucuronide, and 5a-Androstan-3a,17b-diol-17b-glucuronide. The specific associations between SNPs and identified metabolites are listed in Supplementary Table S4. (**B**) The chemical structure of 5a-Androstan-17b-ol-3-one glucosiduronate is shown as a representative example of the metabolites potentially regulated by these transporter genes. (**C**) The SNPs shown are associated with the levels of any implicated metabolites, where purple points refer to the top lead SNP, and other SNPs are represented by points colored by their r2 value. The r2 value, which represents phenotypic variation, is high in these regions and reference SNPs that have previously been analyzed. The nearest mapped genes are shown below each plot. SNPs, which are not in linkage disequilibrium of any significant independent lead SNPs in the selected region, are colored grey.

While our main focus was on the potential shared function of genomic loci in regulating circulating metabolites, the associations between genomic loci and identified metabolites represented potential physiological roles for the implicated genes. The strongest associations in our study were between genomic locus 53 and conjugated sex steroids (Figure 8). This genomic locus contains a cluster of genes in the SLC22 family that are best known for their role in organic anion transport [49]. Recent functional studies have shown that five conjugated sex steroids directly interact with SLC22A24 in vitro, as well as in GWAS [50]. Here, we report that four similar metabolites (Putative_5a-Androstan-17b-ol-3-one glucosiduronate, Putative_Androstan-3-ol-17-one 3-glucuronide, Putative_Androstan-3-ol-17-one 3-glucuronide, and Putative_4-Androsten-17b-ol-3-one glucosiduronate) are associated with the genomic locus containing regions relating to *SLC22A6*, *SLC22A8*, *SLC22A9*, *SLC22A10*, *SLC22A24*, and *SLC22A25*. The strongest associations involve Putative_5a-Androstan-17b-ol-3-one glucosiduronate. The SNPs rs78176967, rs142131421, rs113939203, and rs113497640, which had log-scaled *p*-values between −225 and −280, suggesting a strong functional relationship between one or many of the genes expressed on this locus and this metabolite.

### 3.7. SNPs in Drug Transporter and DME Genes Are Pleiotropic and Linked to Multiple Identified Metabolites

Within our subset of surveyed genes were several that are known to be functionally related to multiple classes of drugs. For example, CYP3A4 is among the most promiscuous of the DMEs with hundreds of drug substrates and dozens of endogenous substrates [51]. Among endogenous molecules detected by our metabolomics approach, we found that genomic locus 41, which contained CYP3A4, CYP3A5, and others in the CYP3A family, was associated with multiple identified metabolites, including bile acids, sex steroids, eicosanoids, and prostaglandins. Furthermore, the multispecific SLCO drug transporters in genomic locus 54 were associated with 62 metabolites, mainly eicosanoids and fatty acids. Genomic locus 70 harbored 16 genes, mainly in the CYP4F family, and was associated with 15 metabolites, mostly eicosanoids and fatty acids. Although multiple types of genes are included in locus 70, such as GPCRs, we expect these associations to be due to functional changes in the CYP4F family. The CYP4F family is heavily involved in the metabolism of fatty acids and their derivatives [52]. The fact that many of the associations in this work have been validated in other studies suggests that the novel associations will prove useful in determining potential metabolic roles for the implicated genes.

### 3.8. Implicated SNPs in Endogenous Metabolism Have Been Reported to Impact Drug Handling

**Figure 9:**
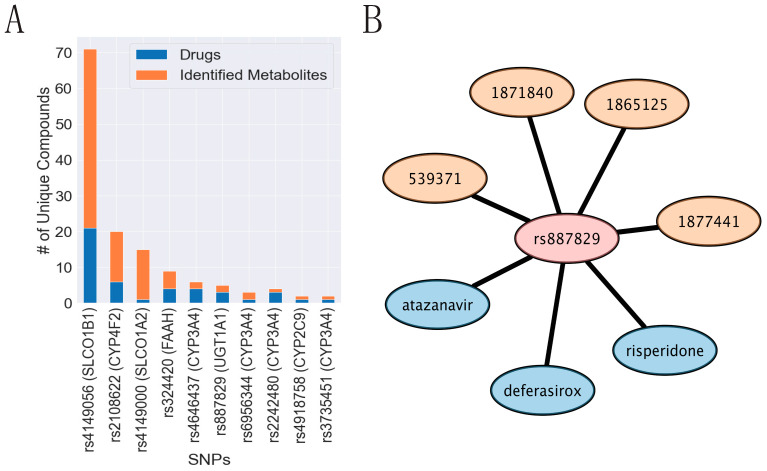
Some SNPs in ADME genes are involved in the regulation of both drugs and endogenous metabolites. (**A**) Certain SNPs are associated with levels of multiple identified metabolites in our study and with several drugs from other independent studies. Rs4149056, an SNP that impacts the function of SLCO1B1, is the most frequent SNP with respect to metabolites and drugs. (**B**) rs887829 (*UGT1A1*) is an example of an SNP that is associated with three different drugs and identified metabolites that are all related to bilirubin (Supplementary Table S4). The SNP is shown as a pink node, the drugs are blue nodes, and the identified metabolite IDs are shown as orange nodes.

As mentioned, many of the SNPs linked to the metabolites in our study have been previously associated with the efficacy or toxicity of different drugs. This begets the question of potential drug−metabolite interactions (DMI). This might also be expected because many of the aforementioned genes are at the interfaces of the Remote Sensing and Signaling (protein) Network and the overlapping ADME protein network. Variant−drug relationships were downloaded from the PharmGKB database and compared to our data to predict potential DMIs. Ten SNPs were present in both our study and the PharmGKB database and were associated with at least one drug (Figure 9). The most common SNP was rs4149056, which is present within the *SLCO1B1* region. In addition to being linked to 50 unique identified metabolites (Supplementary Table S4), this SNP is also associated with affecting 21 unique drugs, including simvastatin, lopinavir, and doxorubicin. Most of the SNP-drug pairs associated with SNPs in our study were present in genomic locus 54, which is consistent with its role in the regulation of endogenous metabolites. Indeed, SLCO1B1/SLCO1A2 are well-known as multispecific drug transporters with a wide array of both xenobiotic and endogenous substrates [53]. In addition to genomic locus 54, genomic locus 70 (containing CYP4F genes) had the second most associations, with 14 unique metabolites and 6 unique drugs. As we discuss below, the use of SNPs linked with both drug handling and endogenous metabolism is likely to be useful for predicting clinically relevant drug−metabolite interactions.

## 4. Discussion

The Remote Sensing and Signaling Theory emphasizes the role of multi-, oligo-, and monospecific transporters, enzymes, and regulatory proteins in the homeostasis of endogenous metabolites, signaling molecules, antioxidants, and other small molecules with “high informational content” in bodily fluids and tissues by mediating inter-organ and inter-organismal (gut microbe-host) communication [11]. These transporters and enzymes lead to the availability of these metabolites and signaling molecules in specific tissues and body fluids, often “setting up” the classical signaling events by GPCRs, nuclear receptors, and kinases. Since many of the molecules involved in signaling via cell surface and nuclear receptors are small, polar, bioactive metabolites (e.g., free fatty acids, eicosanoids, bile acids, fatty acid esters of hydroxy fatty acids), we utilized non-targeted LC-MS methods that specifically capture these and other physiologically important molecules [34,35]. This approach also allowed us to explore both known and unknown chemistries of circulating molecules.

Many aspects of Remote Sensing and Signaling Theory are supported in model organisms, including mice and flies [24,25,27,28,54,55,56], and are beginning to be supported in human studies [23,29]. Key to the theory is the development of as comprehensive a parts list as possible—consisting, for instance, of interacting transporters and enzymes with their metabolite substrates. One approach to identifying the Remote Sensing and Signaling (protein) Network has been through the creation and analysis of cross-tissue co-expression networks of multi-, oligo- and monospecific transporters, enzymes, and nuclear receptors [11]. This led to a preliminary gut−liver−kidney Remote Sensing and Signaling (protein) Network involved in endogenous metabolism that included, as hubs, many well-known SLC and ABC “drug” transporters and DMEs among its ~600 nodes. Thus, it was not surprising that there was similarity and overlap with a smaller network that specifically integrated ADME proteins [11]. However, it is important to keep in mind that the apparent physiological objective of the Remote Sensing and Signaling Network is the mediation of endogenous small molecule homeostasis, while a large part of what the ADME network is presumed to mediate is the metabolism and elimination of drugs.

That said, a major goal here was to define the metabolites and signaling molecules regulated or modulated by multi-, oligo- and monospecific transporters and enzymes in this Remote Sensing and Signaling Network. However, because of the considerable overlap in proteins of the Remote Sensing and Signaling Network and the ADME protein network, it was possible to consider whether drug−metabolite interactions might occur at the interfaces of the two networks [23].

Determining substrates of transporters or enzymes is typically done with in vitro assays or in vivo animal experiments [57]. In silico methods using experimental or predicted protein structures have also been used to predict potential substrates, most notably for enzymes [58,59,60]. Unfortunately, for membrane-bound transporters, there are comparatively few crystal structures available, so protein-based predictions are more difficult to generate [33,61]. GWAS or targeted SNP-association studies in tandem with metabolomics represent another method for determining potential small molecules that may interact with proteins in a direct or indirect way and can suggest a physiological role for these proteins in the modulation of plasma metabolite levels (Figure 1) [62]. Although in vitro or in vivo experiments are required to confirm the interactions, these results can, as described in this study, help broaden the list of potential in vivo interactions of endogenous metabolites with human transporters and drug-metabolizing enzymes. Treating metabolite levels themselves as phenotypes can provide insight into the endogenous metabolic roles of genes and the intermediate processes they may participate in [7,9,10,20].

By uncovering the molecular mechanisms of these proteins in physiological processes, we can improve our understanding of the roles of the hundreds of genes conventionally associated with drug ADME (absorption, distribution, metabolism, elimination), as well as others involved in broader aspects of small molecule homeostasis. We argue their role in endogenous small molecule homeostasis is their major role in humans and other organisms [49,55], but because of the tremendous pharmaceutical and toxicological relevance of these genes, their role in endogenous physiology has largely been neglected. Here, we identified 77 genomic loci containing 284 unique genes (Figure 2) that were associated with the circulating levels of at least one endogenous, polar, bioactive molecule of the kind known to bind signaling receptors on the cell surface and in the nucleus.

Many of the surveyed genes are known to play a major role in drug metabolism and work together along the gut−liver−kidney axis (Figure 3) [56,63,64]. Typically, drugs are absorbed and enter the bloodstream via intestinal transporters. They then enter the liver through hepatic transporters, where the majority of enzymatic drug metabolism occurs. The modified compounds are then cleared or re-introduced to the bloodstream by efflux transporters. If the modified compounds re-enter the bloodstream, they are taken up, metabolized by DMEs in the kidney, and ultimately cleared into the urine by renal transporters or re-introduced into the bloodstream. The same occurs for many small polar metabolites, signaling molecules, antioxidants, nutrients, natural products, gut microbe-derived metabolites, and vitamins. Thus, the remote communication between proteins expressed across these and other organs via small molecules is crucial to the regulation of endogenous metabolism and crosstalk along organ axes or organ systems, as is evident in bile acid and urate homeostasis [15,65]. Defective inter-organ communication involving metabolite transporters, as in the case of *OCTN2*, also considered a drug transporter, can lead to potentially lethal diseases such as Systemic Carnitine Deficiency [66].

While mainly studied for their roles in the ADME of drugs, here we show a number of examples of many of the same ADME proteins jointly contributing to the regulation of a single endogenous metabolite or multiple metabolites. As we have shown, this could involve as many as four transporters and/or enzymes (Figure 4 and Figure 5, Table 1) of the Remote Sensing and Signaling Network potentially overlapping with drug-handling proteins in the ADME network regulating a single metabolite. For example, among unidentified, unique metabolites, five metabolites were associated with four distinct loci. In addition, there were 79 metabolites associated with 3 distinct loci, including 25 distinct combinations of loci. Although most of these metabolites have yet to be fully defined in terms of chemical identity, the loci that influence their circulating levels include multi-, oligo-, and monospecific transporters and enzymes, including well-known drug-handling proteins. For instance, among the identified metabolites, the eicosanoid EIC_311 was associated with SNPs in or relating to the *SLCO1B1*, *CYP3A5*, and *SULT2A1* genes, which are, respectively, a Food and Drug Administration (FDA) highlighted transporter, a Phase I DME and a Phase II DME, all on separate chromosomes (Figure 7). These genes are heavily involved in ADME and implicated in remote sensing and signaling via co-expression analysis and/or in vitro interactions with drugs and metabolites [11]. Understanding the full extent of the role of these genes can also help better understand drug−metabolite interactions (DMIs). DMIs are often ignored in reference to drug side effects and adverse drug reactions, which can potentially be mitigated through better dosing of drugs, so as to not overly perturb the Remote Sensing and Signaling Network involved in small molecule homeostasis across cells, tissues, organs, and organ systems.

While most of the SNP-metabolite associations involved unidentified metabolites, the 98 identified metabolites and their associations with specific SNPs include well-known physiological protein−metabolite interactions (Figure 6). For example, the *UGT1A* locus, which encodes multispecific enzymes involved in Phase II drug metabolism, is also known to modify bilirubin and mutated in human Gilbert’s Syndrome, and that interaction is reflected in our results [67]. Likewise, *SLC22A9/10/24/25*, which appears to be relatively monospecific or oligospecific in one of the SLC22 transporter subgroups [49], was associated with conjugated sex steroids (Figure 8). The role of SLC22A24 in human steroid metabolism and disease has been previously reported [50]. The *CYP3A*, *CYP4F*, and *CYP2C* genes, including multispecific and oligospecific enzymes, are known to generate and degrade signaling eicosanoids and fatty acids, which is reflected in our results here [68,69,70,71]. The multispecific hepatic “drug” transporter OATP1B1 (SLCO1B1), associated with statin myopathy [72], also had several associations with a wide array of small molecules, including eicosanoids, bile acid conjugates, and fatty acids, which is consistent with its known function [73]. FAAH, an enzyme that might be considered oligospecific, is known to modulate the levels of endocannabinoids in tissues, and in this study, we show it also influences the levels of endocannabinoids in plasma [74].

These existing relationships suggest that many unexplored associations between SNPs and identified metabolites may be of great physiological and clinical importance. Among the unexplored relationships with no existing literature to date are those between steroid 5-alpha reductase 2 (*SRD5A2*) and xanthine dehydrogenase (*XDH*) with Allo_Tetrayhydrocortisol in genomic locus 11, the *SLC17* family (transporters of phosphate and other organic anions) with acetyltryptophan in genomic locus 32, and several others. Although we are able to associate a gene family with a class of metabolites, more in-depth studies would be required to confirm the mechanistic relationship between these proteins and metabolites, as well as their joint role in regulating certain metabolic pathways. The identification of the very large number of unnamed metabolites will also allow the design of more functional assays to better define the metabolic role of drug transporters and DMEs and their potential role in DMIs.

The SNPs in genes that are not known to be functionally related to the ADME of drugs or the handling of endogenous metabolites indicate that certain SNPs can indirectly impact the levels of plasma metabolites independent of transport and enzymatic activity. Of the three genomic loci (8, 49, and 56) that do not contain any transporters or enzymes, each has a different potential mechanism for regulating the levels of circulating compounds. The polycystic kidney disease 2-like 1 (*PKD2L1*) gene in genomic locus 49 was linked to 91 metabolites, including 3 named eicosanoids. This gene codes for a calcium channel that is involved in signaling, development, and taste, yet, its direct association with any polar bioactive molecules has yet to be reported [75]. It is expressed in numerous tissues, and the relatively large number of unique metabolites it is associated with suggests that general calcium signaling can have important consequences on the plasma metabolome. Genomic locus 56 contained *HNF1A* (hepatocyte nuclear factor 1 alpha), a nuclear receptor activated by signaling ligands, *HNF1A-AS1,* and *C12orf43*. The open reading frame gene is understudied, but HNF1A and HNF1A-AS1 play roles in transcriptional regulation. Indeed, HNF1A regulates many ADME-related genes in metabolically active organs and thus can impact circulating metabolite levels (amino acids, bicarbonate, sugars) [76,77]. Genomic locus 8 contains *NOS1AP*, a gene that binds to NOS1 for signaling purposes [78]. We examined SNPs in the *NOS1* gene but found no significant metabolite associations. This suggests that NOS1AP, perhaps through the regulation of NOS1-mediated signaling, can modulate more complex interactions that ultimately lead to altered levels of plasma metabolites.

The field of pharmacogenomics is expected to play a major role in personalized medicine in the future, as drug administration and dosage can be more appropriately determined with knowledge of a patient’s genome [79,80]. Many drugs are taken up into the liver by drug transporters (e.g., SLCO family) and then metabolized by Phase I and Phase II DMEs before being eliminated through drug transporter-mediated mechanisms, such as members of the SLC22 family in the kidney. It is important, however, to understand the potential metabolic dysregulations that can stem from existing drugs and entities in the drug development pipeline.

Drug targets differ depending on the intended function, but the proteins involved in the ADME processes overlap greatly with those regulating key processes in endogenous physiology (e.g., bilirubin metabolism, eicosanoids, bioenergetics). Indeed, the Remote Sensing and Signaling Theory argues that drugs often “hijack” endogenous pathways involved in remote organ communication and gut microbe-host communication. Thus, common adverse drug reactions, drug side effects, and drug-induced metabolic diseases may be caused by the competition between drugs and metabolites at the level of so-called drug transporters and drug metabolizing enzymes involved in key biochemical pathways. By comparing the previously determined role of SNPs via the PharmGKB database, we related our analysis to potential drug-metabolite interactions. For example, the rs4149056 SNP in the *SLCO1B1* gene affects drug response, as well as several bioactive molecules. If a patient has this SNP, treatment with a drug impacted by this SNP may exacerbate the metabolic consequences. Within our dataset, we identified 10 SNPs with evidence of potential drug−metabolite interactions (Figure 9). As knowledge of the role of ADME genes in endogenous metabolic processes increases, more will likely be identified.

It is worth emphasizing again that the untargeted metabolomics approach used here focused on small, polar, bioactive metabolites, both identified and unidentified, likely to interact with GPCRs, nuclear receptors, and other signaling proteins—and that they were significantly associated with SNPs in multiple distinct genomic loci. We have also presented Remote Sensing and Signaling Theory as a framework for understanding communication between organs through the regulated expression and function of multispecific, oligospecific, and (relatively) monospecific proteins, such as drug transporters, drug-metabolizing enzymes, and their relatives [11,81,82]. The broad substrate specificity of “drug” transporters and “drug” metabolizing enzymes mainly refers to pharmaceutical products—often with very different structures and mechanisms of action, but this multi-specificity likely also applies to endogenous metabolites, as is clear with the organic anion transporters (OATs), SLC22A6 and SLC22A8 [19]. It is useful to note here that oligospecific and monospecific close relatives of the well-known drug transporters (OATs and organic cation transporters (OCTs)) are strongly implicated in the handling of metabolites like urate (SLC22A12) and carnitine (SLC22A5, SLC22A15/16). This fact emphasizes a main concept in the Remote Sensing and Signaling Theory—that multi-, oligo-, and monospecific transporters and enzymes work within and between organs to optimize endogenous metabolism in cells, tissues, organs, and multi-organ systems [18,19,83].

## Figures and Tables

**Table 1:** Summary statistics of surveyed participants. Participants are from the Framingham Offspring Cohort Exam 8.

Category	Value
Participants	2886
Men	1315
Women	1571 (54.4%)
Age	66 ± 9 years
Body Mass Index (BMI)	28.3 ± 5.4 kg/m^2^

**Table 2:** Five of 7326 unidentified metabolites are associated with four unique combinations of genomic loci. These metabolites are associated with four unique combinations of genomic loci. The mass-to-charge ratio (MZ) and the retention time (RT) of each of the five metabolites are listed and indicate that metabolites 1272586 and 1291919 are likely to be very similar compounds. Genomic loci 28 (*ACSL6*), 29 (*SLC22A4/5*), 46 (*SLC16A9*), and 54 (*SLCO1B1/3/7*, *SLCO1A2*) are associated with more than one metabolite, and genomic loci 4 (*SLC44A5*), 15 (*UGT1A6/7/8/9/10*), 19 (*UGT2B*), and 31 (*ECI2*) appear only once. The full list of genes associated with each locus is presented in Supplementary Table S2.

Genomic Locus 1	Genomic Locus 2	Genomic Locus 3	Genomic Locus 4	mtb	MZ	RT
4	28	29	54	1,380,594	284.2233	4.248833
15	19	48	54	1,116,529	607.3553	3.428667
28	29	46	54	1,272,586	282.2076	3.878833
28	29	31	46	1,291,919	282.2085	3.959
28	29	46	54	1,592,026	310.2399	5.155334

**Table 3:** Eight identified metabolites are associated with two distinct genomic loci. Only one identified metabolite, an eicosanoid identified as EIC_311, was associated with three distinct loci, but eight others were associated with two, suggesting a more specific regulation. Some metabolites are presented twice because their identities are expected to be the same despite minor differences in their MZ or RT values.

Identity	Genomic Locus 1	Genomic Locus 2	MZ	RT
Eicosanoid_13,14-dihydro-15-keto-tetranor-PGE2 [M-H]	3	32	297.1744	1.816083
Eicosanoid_12-HHTrE [M-H+Acetate]	47	54	339.2178	3.669167
Eicosanoid_12-HHTrE [M-H+Acetate]	47	54	339.2197	3.766292
Putative_N-Oleoyl-L-serine	2	54	368.2847	6.353979
Putative_N-Oleoyl-L-serine	2	54	368.286	6.253
Endocannabinoid_Oleoyl Ethanolamide [M-H+Acetate]	2	70	384.3096	6.401
Endocannabinoid_Oleoyl Ethanolamide [M-H+Acetate]	2	70	384.3174	6.479625
Putative_Androstan-3-ol-17-one 3-glucuronide	41	53	465.2491	2.152167
Putative_Androstan-3-ol-17-one 3-glucuronide	41	53	465.2492	2.0535
Putative_Androstan-3-ol-17-one 3-glucuronide	41	53	465.2498	2.2015
Putative_5a-Androstan-3a,17b-diol-17b-glucuronide	19	53	467.2574	2.035
Putative_5a-Androstan-3a,17b-diol-17b-glucuronide	19	53	467.2634	1.89625
Putative_Chenodeoxycholic acid 24-acyl-b-D-glucuronide	19	54	567.3177	2.890625
Putative_Chenodeoxycholic acid 24-acyl-b-D-glucuronide	19	54	567.3182	2.76575
Putative_1,3,5(10)-Estratrien-3,17b-diol diglucosiduronate	41	54	623.3406	2.713333
Putative_1,3,5(10)-Estratrien-3,17b-diol diglucosiduronate	41	54	623.342	2.58075
Putative_1,3,5(10)-Estratrien-3,17b-diol diglucosiduronate	41	54	623.3441	2.540667

## Data Availability

The data presented in this study are available in Supplementary Materials.

## Supplementary Materials

The following supporting information can be downloaded at: https://www.mdpi.com/article/10.3390/metabo13020171/s1, Supplementary Figure S1: Five unique metabolites were associated with 4 unique loci, 79 unique metabolites were associated with 3 unique loci, 606 unique metabolites were associated with 2 unique loci, and 6636 metabolites were associated with one locus. Supplementary Table S1: Original list of genes selected for targeted SNP associations. These genes include transporters, enzymes, and related proteins that are known to handle small molecules or related to proteins that do. Supplementary Table S2: Genomic locus and gene assignments that the detected SNPs associated with circulating metabolites map to. Genes are listed by their HUGO gene nomenclature committee (hgnc) symbols. ENSEMBL gene IDs are also listed in a separate column. The start and end position of each gene on the chromosome is listed. All positions come from the GRCh37 build. Supplementary Table S3: All unique statistically significant SNP-metabolite associations detected are listed. Snp: Single nucleotide polymorphism. Snp_cpra: Single nucleotide polymorphism with chromosome position and reference and alternate alleles. mtb: Metabolite id. MZ: Mass to charge ratio. RT: Retention time. chr: Chromosome. pos: Position. ref: Reference allele. alt: Alternative allele. pvalue: *p*-value indicating statistical strength of association between SNP and metabolite. beta: Beta coefficient for fit. se: Standard error. alt_freq: Alternative frequency. Supplementary Table S4: All unique statistically significant SNP-metabolite associations involving identified metabolites. Identity: Putative endogenous metabolite with the unique combination of MZ and RT. Genomic Locus: Assigned genomic locus listed in Supplementary Table S2. Snp: Single nucleotide polymorphism. Snp_cpra: Single nucleotide polymorphism with chromosome position and reference and alternate alleles. mtb: Metabolite id. MZ: Mass to charge ratio. RT: Retention time. chr: Chromosome. pos: Position. ref: Reference allele. alt: Alternative allele. pvalue: *p*-value indicating statistical strength of association between SNP and metabolite. LogP: Logarithmic value of *p*-value. beta: Beta coefficient for fit. se: Standard error. alt_freq: Alternative frequency. Mass_error: Error bars for accuracy of mass to charge ratio. RT_error: Error bars for accuracy of retention time. SMILES (if available): For each entry in identity, the isomeric SMILES sequence is listed. For metabolites with unknown identity (e.g., EIC_311), NA is listed.
