# Supplementary figures and images for: Regulation of Human Endogenous Metabolites by Drug Transporters and Drug Metabolizing Enzymes: An Analysis of Targeted SNP-Metabolite Associations
Source: Metabolites. 2023 Jan 24;13(2):171. doi: 10.3390/metabo13020171 (PMC9958903; doi:10.3390/metabo13020171)

Number of unique metabolites associated with distinct loci

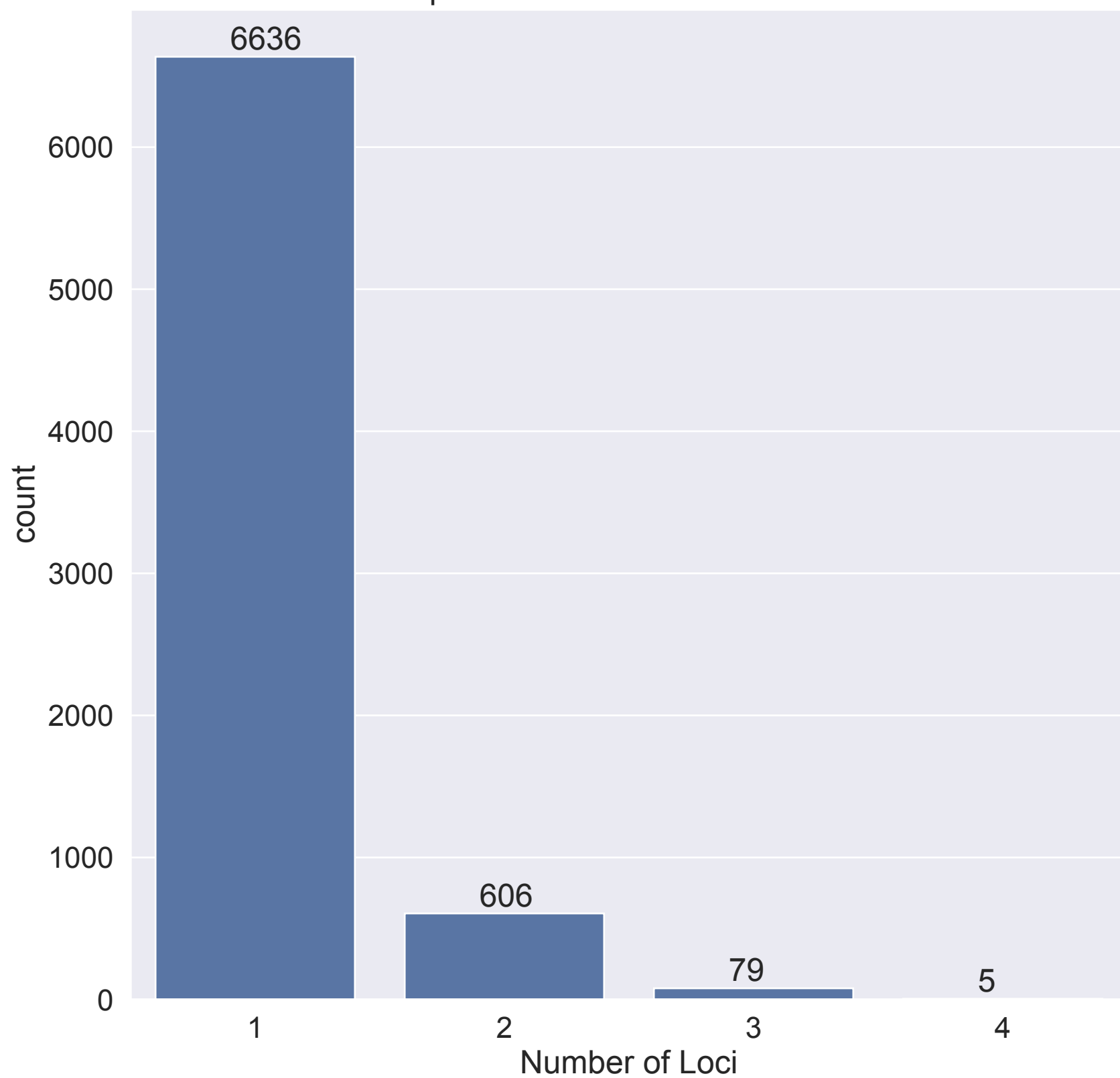

Supplement: Supplementary file 1 [file metabolites-13-00171-s001.zip › 012323_SupplementaryFiles/012323_SupplementaryFigureS1.pdf]
